# An Ovarian Pregnancy in a Patient with a History of Bilateral Salpingectomies: A Rare Case

**DOI:** 10.1155/2015/740376

**Published:** 2015-03-17

**Authors:** Sadia Khandaker, Pranav Chitkara, Eric Cochran, Jed Cutler

**Affiliations:** Department of Obstetrics and Gynecology, King's County Hospital Center, Brooklyn, NY, USA

## Abstract

*Background*. 1 in 200 ectopic pregnancies are true ovarian pregnancies that fulfill the Spiegelberg criteria. Despite being rare, multiple case reports and series have been reported. Few cases have been published in which the event was preceded by salpingectomy. *Case*. The patient is a 32-year-old female who presented to the emergency room with abdominal pain. She was found to be pregnant, despite a history of two previous ectopic pregnancies treated with salpingectomies. Sonography confirmed a left adnexal mass and free fluid. Surgery revealed a ruptured ovarian pregnancy which was also confirmed by pathology. *Conclusion*. This is a case of an ovarian pregnancy in a patient with two previous salpingectomies. It underscores the importance of searching for an ectopic pregnancy in patients with abdominal pain after fertility impairing surgery.

## 1. Introduction

Ectopic pregnancy continues to be the most common cause of first trimester maternal death [1]. While the majority of cases are tubal pregnancies, less than 1% are ovarian or cervical [1]. One in 200 ectopic pregnancies is true ovarian pregnancies that fulfill the Spiegelberg criteria. (1) The tube and fimbria must be intact and separate from the ovary. (2) The gestational sac must occupy the normal position of the ovary. (3) The sac must be connected to the uterus by the ovarian ligament. (4) Ovarian tissue should be demonstrable in the walls of the sac [2]. Despite being rare, multiple case reports and some series of ovarian pregnancies have been reported [1]. However, very few cases have been published in which the event was preceded by bilateral salpingectomy. Here we present a case of an unusual ovarian pregnancy in a patient with a history of bilateral salpingectomies for 2 prior tubal pregnancies [1].

## 2. Case

The patient is a 32-year-old G5 P1031 whose last menstrual period was approximately 2 weeks prior to presentation. She presented to the emergency room with the complaints of abdominal pain, sporadic dysuria, and difficulty passing stool. She denied vaginal bleeding, chest pain, shortness of breath, or palpitations.

The patient's obstetric history was significant for two ectopic pregnancies. She underwent an exploratory laparotomy and a right salpingectomy for a right tubal pregnancy in Jamaica, West Indies in 2007. Subsequently, she had a laparoscopic left salpingectomy for a tubal pregnancy at our institution in 2009. The pathology report from 2009 confirmed a left tubal pregnancy.

**Figure 1:**
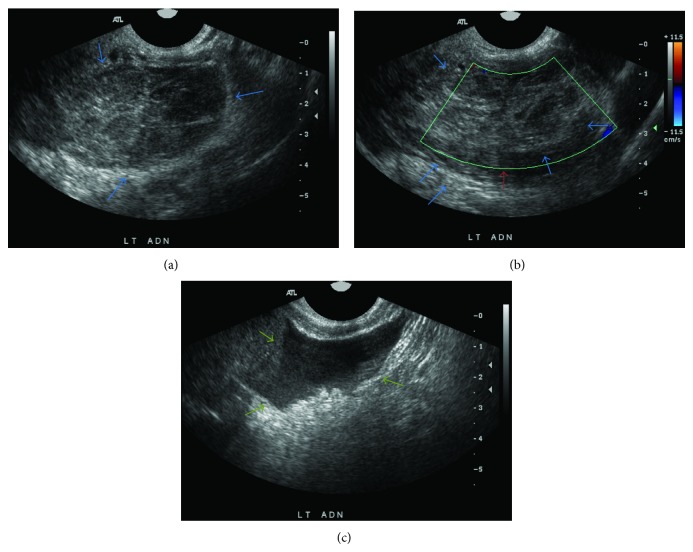
Sonographic images of the left adnexal region with and without color Doppler: large left adnexal mass (blue arrows), measuring approximately 6.3 × 3.9 cm, with heterogeneous echogenicity and no appreciable flow on color Doppler (red arrows). There is a moderate amount of free fluid (green arrows). Left ovary not definitively visualized [3].

The patient had informed the emergency staff that she could not be pregnant, as she had both tubes previously removed. Despite this, a urine pregnancy test was ordered as part of the emergency room's routine lab work, and it was found to be positive. Her serum beta human chorionic gonadotropin (beta-hCG) level was 880 milli-international units/mL. Additional hematologic parameters were within normal limits (WBC count 8.9 hematocrit 32.7) and the comprehensive metabolic panel was unremarkable (sodium 139 potassium 3.7 bicarbonate 104 chloride 22 BUN 9 creatinine 0.93 glucose 95 mg/dL, liver function AST/ALT 16 and 15). Transvaginal sonography was performed (Figures 1(a), 1(b), and 1(c)) and revealed a left adnexal mass measuring 6.3 × 3.9 centimeters (Figure 1(a)) with no Doppler flow (Figure 1(b)) and a large amount of complex free fluid in the pelvis (Figure 1(c)).

**Figure 2:**
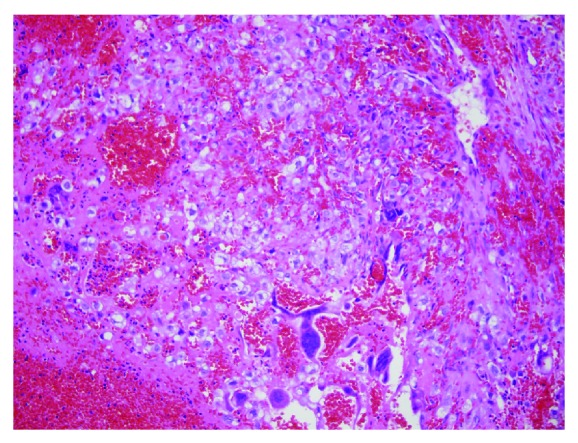
Ovarian cyst with hemorrhage, decidua, and trophoblastic tissue [4].

The impression was a ruptured ectopic pregnancy and surgical intervention was recommended. The patient consented and laparoscopy was performed. Upon entry into the abdomen, 1000 milliliters of clotted and liquid blood was found. The stumps of both fallopian tubes were present and the right ovary was found to be unremarkable. In the region of the left adnexa, dense adhesions were found. Laparoscopic adhesiolysis revealed diffuse involvement of the large bowel with the incorporation of the left ovary into the mass. Active bleeding was noted from the exposed portion of the ovarian tissue and the decision was made to convert to laparotomy. Further dissection at laparotomy allowed full visualization of the ovary. Two 1 cm defects were identified, one of which was actively bleeding and the other spilling grossly identifiable trophoblastic material surrounding a gestational sac. This tissue was easily removed and submitted to pathology for permanent section. Bleeding points on the ovarian surface and in the area of implantation were controlled with suture ligation. The ovary was otherwise normal and was left in situ. Postoperatively, the patient recovered without any adverse events and was followed with serum beta-hCG levels which declined appropriately.  Figure 2 shows the pathologic analysis of the specimen from the procedure; it confirms the diagnosis of an ovarian pregnancy.

## 3. Comment

We present a case of an ovarian pregnancy that occurred in a patient with a history of two previous tubal pregnancies, both of which were treated with salpingectomy. As the antecedent tubal pregnancy was diagnosed and treated at our institution, the pathology report and glass slides were available for review. This review confirmed the original diagnosis and provided a clinical and surgical correlation with the intraoperative findings of the absence of tubes and fimbriae. While the Spiegelberg criteria stipulates that the distal tube and fimbria must be identified to confirm the diagnosis of ovarian pregnancy, the surgical absence of the tube further strengthens the pathologic diagnosis of an ovarian ectopic in this case. This is especially relevant as the purpose of the criteria was to exclude ectopics originating in the tube that simply appeared to incorporate ovarian tissue. The absence of the tube rules out the possibility that the final diagnosis is an “overdiagnosis.” The remaining pathologic criteria published by Spiegelberg were satisfied.

The clinical presentation of an ovarian pregnancy is similar to other ectopic pregnancies in general. This patient presented classically. The history of the present illness and the findings on laboratory evaluation and imaging studies were typical of an ectopic pregnancy. The clinical course, including the outcome of the surgery and the postoperative recovery, was unremarkable. What makes this case unique is the history of bilateral salpingectomy.

Using PubMed, we performed a search using the following key words: “ovarian,” “pregnancy,” “recurrent,” and “ectopic” and did not find another case of an ovarian pregnancy in a patient with a history of two previous tubal pregnancies. There was a case of recurrent ectopic pregnancies in the remnant of a tube following salpingectomy [5]; however, no reports of ovarian pregnancy in this population were found. A second case report described an ovarian pregnancy through in vitro fertilization following bilateral salpingectomy for hydrosalpinx, and fistula formation from the cornua was hypothesized as the etiology [6]. We theorize that the rarity of our event is attributable to the alignment of three rare events: pregnancy in a patient without fallopian tubes, pregnancy, and ovarian implantation. One may posit that the absence of fallopian tubes increases the likelihood that if an ectopic pregnancy occurs, it will, by process of elimination, occur more frequently in the ovary. In reality, the fact that pregnancy occurs at all, in a patient without fallopian tubes, outweighs the competing premise.

The incidence of an ovarian ectopic pregnancy and ectopic pregnancy in general has been rising over the past 20 years. A consensus has emerged that attributes this finding to increases in the risk factors for ectopic pregnancy. These factors are climbing rates of ascending pelvic infection, the trend in delaying childbirth into the 4th and 5th decades, and greater availability and application of assisted reproductive technologies. Of note, the patient presented in this case did not have a history of pelvic inflammatory disease, was young, and did not use fertility drugs.

## 4. Conclusion

This is a rare case of an ovarian pregnancy in a patient with a history of two previous tubal pregnancies and two previous salpingectomies. This case underscores the importance of maintaining vigilance in the search for ectopic pregnancy in patients presenting with abdominal pain after fertility impairing surgery.
